# Corticosteroid-sparing topical treatment with cyclosporin for juvenile keratoconjunctivitis

**DOI:** 10.1038/s41598-025-85256-z

**Published:** 2025-02-08

**Authors:** Amarilla Barcsay-Veres, Anita Csorba, Illes Kovacs, Laszlo Tothfalusi, Otto Alexander Maneschg

**Affiliations:** 1 Department of Ophthalmology, Semmelweis University, Maria Str. 39, Budapest, H-1085 Hungary; 2 Department of Pharmacodynamics, Semmelweis University, Budapest, Hungary

**Keywords:** Vernal keratoconjunctivitis, Blepharokeratoconjunctivitis, Cyclosporin, Corneal fluorescein staining, Steroid-sparing, Eye diseases, Inflammatory diseases, Inflammation

## Abstract

Ocular surface inflammation due to allergy and blepharitis can lead to corneal complications and visual impairment. The aim of this study is to evaluate the efficacy of a cyclosporin 0.1% topical treatment achieving steroid-sparing. Eighty pediatric patients with moderate and severe vernal and blepharitis-related keratoconjunctivitis were included. Symptoms (photosensitivity, itching, discharge, tearing), signs (corneal fluorescein staining, papillary hypertrophy) and patients’ subjective assessment were evaluated during a 6-month follow-up. At the follow-up, all patients treated with topical cyclosporin showed a significant improvement in all subjective symptoms and objective signs (*p* < 0.001). The total number of courses of rescue steroids courses decreased from 3.71 ± 1.72 to 0.25 ± 0.49 at month 3 and to 0.13 ± 0.38 dropping bottle at month 6 (*p* < 0.001 at both time points). The 96.1% of the allergic cohort and 96.4% of the blepharitis cohort experienced a satisfactory good or rapid and good effect during the 6-month follow-up. The probability of needing rescue corticosteroids increased with an odds ratio of 1.98, (95% CI: 1.19–3.28, *p* = 0.008) for each unit increase in Oxford score when analysing the whole cohort. Topical cyclosporin seems to be very effective reducing the number of recurrences of corneal involvement and the need for steroid treatment.

## Introduction

Chronic, non-infectious ocular surface inflammation affects quality of life and psychosocial wellbeing as early as 4–7 years of age, often impairing learning and physical and mental development^1–8^. The ocular surface is often irreversibly damaged in a vicious cycle, regardless of the cause of prolonged inflammation in the young and adolescent population. Permanent visual impairment may result from persistent ocular surface inflammation alone^4,9–15^ or from prolonged treatment with corticosteroid drops^7,9,16–18^. The prevalence of vernal keratoconjunctivitis (VKC) is underestimated^3,19,20^, though corneal involvement, e.g. punctal keratitis, ulceration, “shield” ulcer (plaque), corneal scarring, thinning is present in 25–50% of cases^9^. In addition, certain keratoconjunctivitis may present fibrotic remodelling of the eyelids^21^. Associated otorhinolaryngological, pulmonological, dermatological or psychological disorders can often be treated only by a multidisciplinary approach^2,17,22^.

In cases of blepharokeratoconjunctivitis (BKC), the inflammation of the eyelid secondary destroys the ocular surface leading to irreversible visual impairment via corneal scarring (irregular astigmatism, amblyopia)^23^. Furthermore, rosacea, which may present with the absence of dermal centrofacial erythema and telangiectasia in younger age groups, is underdiagnosed as well. However, associated blepharokeratoconjunctivitis has been reported in 5–33% of cases^4,18,24^ and is often linked with allergic and endocrine systemic pathologies and psychosocial problems^6,14,15^.

The pathology of VKC and BKC is characterised by persistent inflammation, which is directed towards tissue destruction. This may be driven in VKC by an IgE-mediated immune response^3,11,25^, however, both VKC and BKC are also influenced by the activation of neutrophils and eosinophils, with an inflammatory response that is dominated by corneal keratocytes and conjunctival fibroblasts. This pathology is driven by a predominantly T-cell-mediated process^3,26^. In addition, infectious components^7,27^ or immune process dysregulation^4,5,13,18^ may also be involved.

The aforementioned ocular surface inflammatory diseases share an immunological, vascular, and neural dysregulation in their etiology^3,4,7,27,28^, the control of which is essential for the prevention of visual deterioration resulting from tissue damage. In accordance with Bonini’s clinical grading system, VKC with corneal damage is managed with topical cyclosporin-A (CsA) and/or pulsed high-dose topical steroids^20,29,30^. Similarly, in cases of lid pathology-related keratitis, the therapeutic approach is topical administration of corticosteroid drops, which may potentially result in harmful ocular or even metabolic side effects associated with their use in a paediatric cohort^7,9,16–18^.

The 1 mg/ml (0.1%) cyclosporin-A oil in water cationic nanoemulsion (CsA-CE, Ikervis^®^) eye drop is indicated for the long-term anti-inflammatory treatment of ocular surface inflammation. It has been shown to have a beneficial effect in cases of severe dry eye (EMA/56994/2015)^31–34^, VKC^29,34,35^ and BKC^7,28,35^. The CsA-CE eye drop has superior bioavailability compared to conventional CsA drops, with minimal adverse effects apart from a stinging sensation at the time of drop application^35,36^.

It is pertinent to enquire whether the introduction of Ikervis^®^ eye drop treatment impacts patients’ overall well-being, clinical presentation and long-term corticosteroid requirements in young patients with moderate to severe VKC and BKC. Moreover, it would also be beneficial to determine whether there are any differences in the efficacy between these two types of ocular surface inflammation.

## Results

### Study population

In total, 80 patients (52 with VKC and 28 with BKC) completed the study. No patients withdrew early on the reasons of lack of efficacy or intolerable side effects. The demographic and baseline clinical characteristics were comparable between the VKC and BKC groups (Table 1). The mean duration of prior treatment was 2.73 ± 2.16 years, which is a notably lengthy period for both groups (Table 1). The prevalence of moderate to severe corneal involvement was comparable between the VKC and BKC groups. At the baseline assessment, the majority of subjects demonstrated an Oxford scale grade of 1 or 2 (78.8% and 78.5%, respectively) and grade 3 or 4 (21.2% and 21.5% in the VKC and BKC groups, respectively). The mean VKC grading by Bonini was 2.17 ± 1.02 in the VKC group. Patients who were CsA-CE-naïve received a markedly elevated corticosteroid dosage prior to the initiation of CsA-CE therapy (mean 3.71 ± 1.72 dropping bottle in the previous 3 months), with no notable distinction between the severe or moderate VKC and BKC groups (3.91 ± 1.83 vs. 3.38 ± 1.52, *p* > 0.05.).

**Table 1:** Demographic and baseline clinical characteristics of the full set cohort.

	VKC	BKC	Total
Number of cases n	52	28	80
Gender, no. (%)			
Male	39 (75.0)	11 (39.3)	50 (62.5)
Female	13 (25.0)	17 (60.7)	30 (37.5)
Race, no. (%)			
Caucasian no. (%)	52 (100)	28 (100)	80 (100)
Age (yrs)	8.98 ± 3.45	10.12 ± 5.79	9.36 ± 4.50
Age range (yrs)	4–20	4–20	4–20
Years since diagnosis	2.59 ± 1.79	2.99 ± 2.72	2.73 ± 2.16
Oxford scale prior CsA-CE treatment	1.72 ± 1.31	1.86 ± 0.78	1.77 ± 1.15
Oxford scale 1–2 n (%)	41 (78.8)	22 (78.5)	63 (79)
Oxford scale 3–4 n (%)	11 (21.2)	6 (21.5)	17 (21)
Steroid drops dose 3 months prior to CsA-CE*	3.91 ± 1.83	3.38 ± 1.52	3.71 ± 1.72

* Topical steroid treatment given for 3 months prior to the start of treatment with CsA-CE (No. of bottle during the 3 months period: Data: mean ± SD or number (%).

### Subjective and objective changes on the ocular surface during the follow-up period

**Fig. 1:**
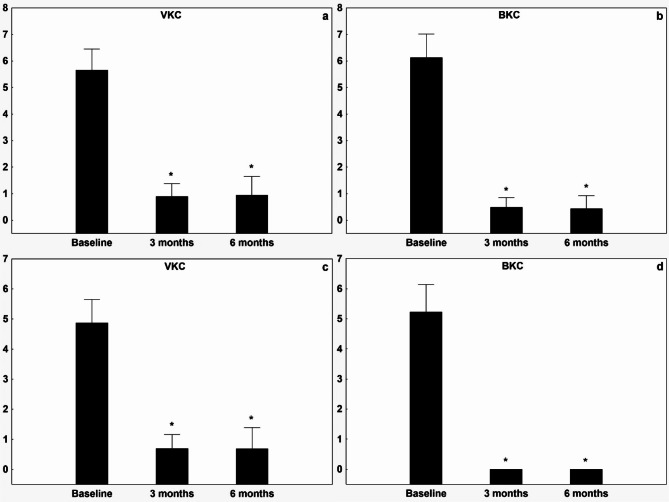
Improvement in symptoms of photophobia and tearing after initiation of CsA-CE treatment at 3 and 6 months compared with baseline in patients with vernal keratoconjunctivitis or blepharitis-related keratoconjunctivitis. Graph showing mean ± SD improvement in the visual analogue scale (VAS) score (0–10) for the symptoms of (**a**) photophobia in the VKC group, (**b**) photophobia in the BKC group. Tearing (**c**) in the VKC group, (**d**) tearing in the BKC group. A decrease in VAS score from baseline indicates improvement. * indicates statistically significant (*p* < 0.05) improvement from baseline.

**Fig. 2:**
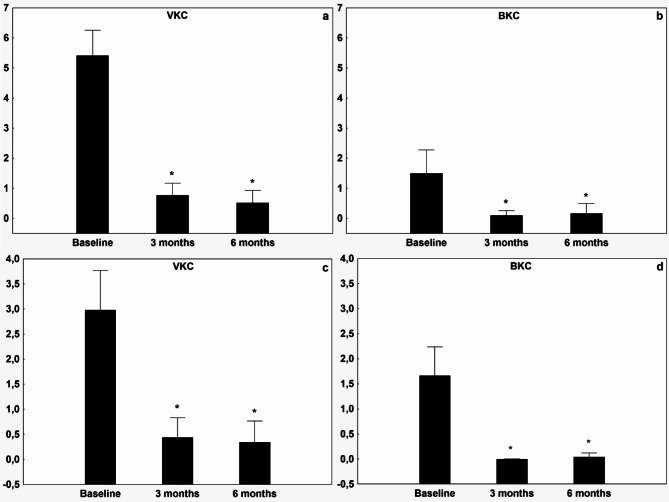
Improvement in symptoms of itching and discharge after initiation of CsA-CE treatment at 3 and 6 months compared with baseline in patients with vernal keratoconjunctivitis or blepharitis-related keratoconjunctivitis. Graph showing mean ± SD improvement in the visual analogue scale (VAS) score (0–10) for the symptoms of itching in the VKC group, (**a**) and in the BKC group (**b**). Discharge in the VKC group (**c**) and the BKC group (**d**). A decrease in VAS score from baseline indicates improvement. * indicates statistically significant (*p* < 0.05) improvement from baseline.

**Fig. 3:**
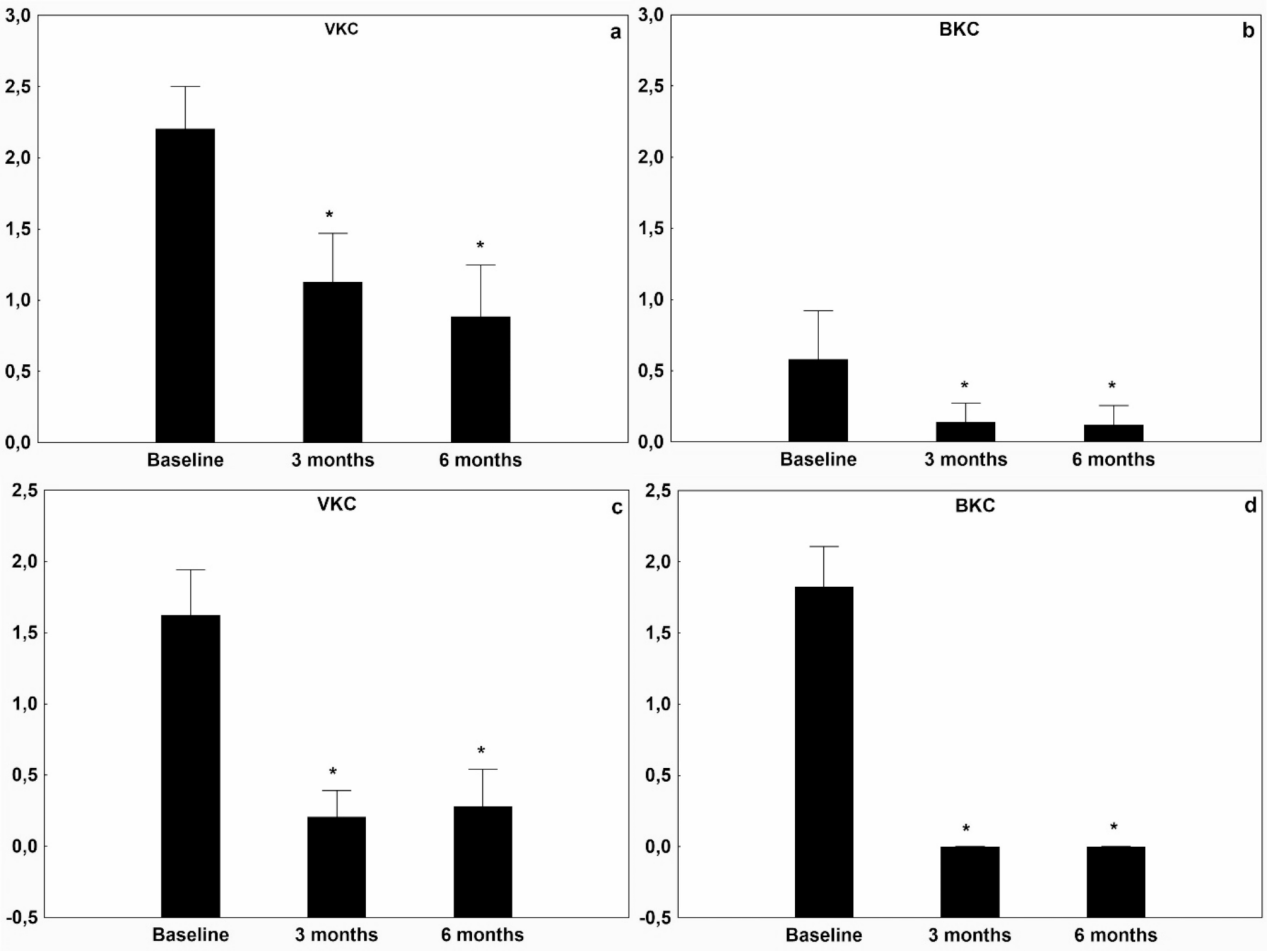
Improvement in objective signs of vernal keratoconjunctivitis and blepharitis-related keratoconjunctivitis after initiation of CsA-CE treatment during the 3-, and the 6-month follow-up compared to baseline values. Graph showing mean ± SD improvement in visual analogue scale (VAS) score (0–5) for Bonini classification of hyperemic and swollen papillae on the tarsal surface of the eyelid in the VKC group (**a**), in the BKC group (**b**). Corneal fluorescein staining [CFS] on the modified Oxford scale in the VKC group (**c**), and in the BKC group (**d**). A decrease in VAS score from baseline indicates improvement. * indicates statistically significant (*p* < 0.05) improvement from baseline.

**Fig. 4:**
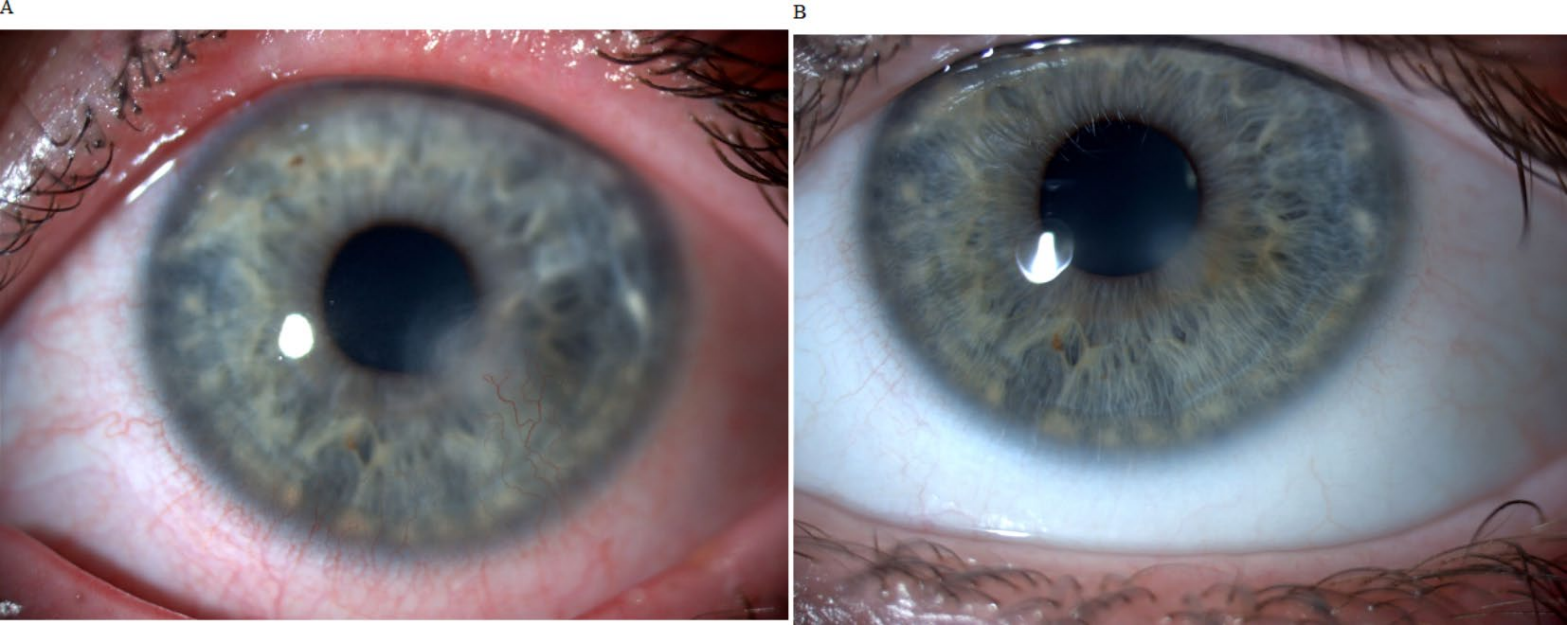
Clinical pictures before and after CsA-CE treatment. (**A**) Clinical picture of a BKC patient before CsA-CE treatment showing lid margin telangiectasia, lipid deposition, conjunctival hyperemia, corneal involvement with neovascularisation and corneal scarring. (**B**) After CsA-CE treatment, the conjunctiva is white and calm. The corneal blood vessels are retracted, with only fine corneal opacities indicating previous corneal inflammation.

The patients’ symptom scores for photosensitivity, tearing, itching, and secretions were all significantly improved in each treatment group (Figs. 1 and 2) over the three- and six-month periods. Moreover, the Bonini score and Oxford scale demonstrated significant improvement across all groups (to 0.71 ± 0.96 and 0.12 ± 0.44 respectively, see Figs. 3 and 4). The treatment effect of CsA-CE drop was highly significant (*p* < 0.001) in all groups (Figs. 1, 2, 3 and 4). All treated groups showed positive and statistically significant improvements regarding the Oxford scale, photosensitivity, discharge and tearing compared to the baseline (control) value. However, there was considerable variation in the Bonini scale and itching parameters across the diagnostic groups, resulting in higher baseline values for the VKC group compared to the BKC group. Furthermore, the improvement in itching and the Bonini score was significantly higher (*p* < 0.001) in the VKC group compared to the BKC group as a result of CsA-CE therapy after three months of treatment.

The subjective assessment carried out by the patients undergoing CsA-CE therapy at the sixth month of follow-up is presented below: The mean overall score was 2.61 ± 0.56, with the majority of patients experiencing a satisfactory good (score 2) or rapid and good effect (score 3). This was observed in 96.1% of the VKC and 96.4% of the BKC cohort. The effect of CsA treatment on subjective symptom scores was more pronounced in the BKC group than in the VKC group (2.85 ± 0.45 vs. 2.49 ± 0.57, *p* = 0.005). The treatment response was not dependent on baseline photosensitivity (*p* = 0.18), discharge (= 0.83) and tearing (*p* = 0.67).

A summation of the subjective and objective clinical outcome scores revealed no significant difference in improvement over the three-month treatment period between the VKC and BKC groups (15.62 ± 7.05 vs. 19.24 ± 1.81, *p* = 0.09).

Patients tolerated CsA-CE therapy very well, with 9.2% of patients reporting mild burning or discomfort when instilling CsA-CE drops.

### Steroid sparing effect of CsA-CE treatment

Following the introduction of CsA-CE therapy, 71.25% of patients exhibited a marked reduction in the necessity for steroid drops over the course of the six-month follow-up period. In comparison with the three months of topical steroid treatment that preceded the introduction of CsA-CE therapy, the entire study cohort exhibited a notable reduction in the quantity of steroid eye drops required at the completion of the three- and six-month follow-up periods (from 3.71 ± 1.72 to 0.25 ± 0.49 bottles after three months, and to 0.13 ± 0.38 bottles after six months, both *p* < 0.0001). Both the VKC and BKC groups exhibited a significant reduction in the necessity for topical steroid drops following a three-month period compared to the baseline (VKC group 0.33 ± 0.55 vs. 3.89 ± 1.81, BKC group 0.11 ± 0.32 vs. 3.44 ± 1.57 respectively, both *p* < 0.0001). The steroid-sparing effect was maintained until the sixth months of CsA-CE treatment.

The proportion of patients who were able to control their disease without any topical rescue steroid therapy during the six-month CsA-CE treatment was 55.72%, in the VKC group and 85.7% in the BKC group, with a statistically significant difference between the two groups (*p* = 0.03).

Finally, the probability of needing rescue corticosteroids increased with an odds ratio of 1.98, (95% CI: 1.19–3.28, *p* = 0.008) for each unit increase in Oxford score analysing the whole cohort.

## Discussion

In our study, initiation of treatment with CsA-CE significantly reduced subjective symptoms, objective signs and the need for topical corticosteroid treatment in the VKC and BKC groups. The main findings are consistent with previous studies on the use of CsA-CE^28,31,35,36^. Randomised controlled trials of CsA 0.05–2.0% as a replacement for corticosteroids are available^28,31,35,36^. The Vektis (VErnal KeratoconjunctiviTIs Study) trial^29^ and a post-marketing study of CsA 0.1% aqueous ophthalmic solution^37^ included mostly patients with severe keratitis (75–90% with baseline Oxford grade 4) and the treatment effect was mainly driven by the CFS score. Desmukh et al.^38^ treated allergic patients with Ikervis, and Doan et al.^39^reported the use of cyclosporin 2% in 11 patients with BKC with good local tolerability and no recurrence during long follow-up. All studies^29,33,38–40^ showed efficacy in improving signs and symptoms of VKC and BKC, but the effect of the introduction of CsA on the need for and extent of steroid drop use has not been extensively studied.

Non-infectious ocular inflammation typically persists for years^3,9,10,19,41^ and is frequently underdiagnosed^2,4,7,15,18^. The use an of ineffective or side-effect inducing treatments is a cause for concern as it may lead to permanent visual impairment^7,9,16–18^. Our study group presented with symptoms of ocular surface inflammation for a period of six to 120 months. Of the participants, 58% were aged 7 years or younger, indicating a high likelihood of continued active chronic ocular surface inflammation over the next 5 years. If learning, sports or other activities are restricted during this crucial period, there is an increased risk of amblyopia and mental health problems^1,20,23^. It is therefore imperative that early, effective treatment with a minimal side-effect profile is made available.

A limitation of the Vektis study^29^ was the inclusion of only severe VKC patients, but our data reflect that even in moderate cases, topical corticosteroid administration prior to CsA initiation was significant (3.71 ± 1.72 drop bottles in the 3 months prior to CsA-CE treatment). Our cohort had moderate to severe corneal lesions (63% Oxford grade 1–2, 17% Oxford grade 3–4), providing new data on the importance of CsA-CE treatment in significantly reducing the topical corticosteroid burden in the paediatric cohort of VKC and BKC patients with moderate to severe corneal involvement. Our study reports real-world experience, and the 6-month timeframe allows evaluation of the entire pollen season. Given the prolonged nature of ocular surface inflammation, the risk of corticosteroid side effects in this population is not negligible. Reducing the need for corticosteroids is likely to improve compliance and the side effect profile.

The introduction of CsA-CE treatment at a dose of 1–4 drops per day resulted in a favourable evolution of clinical scores and reduced corticosteroid use in both the BKC and VKC groups. The CsA-CE formulation allows for improved ocular delivery via increasing the residence time on the ocular system^42^ and performs similar efficacy to the 2% CsA dose^32,39^. As young patients or their caregivers may not be able to cope with the challenge of instillation 4 times a day for long months, compliance can be improved by reducing the dose in the knowledge that efficacy will not be lost. Ikervis^®^ uses cetalkonium chloride (CKC, a cationic surfactant) to positively charge nanoemulsion droplets in the formulation to prolong intraocular drug retention. The use of oily matrices and surfactants can be associated with side effects such as ocular pain or irritation, but in our study population these adverse effects were observed at levels consistent with the safety profile in the Summary of Product Characteristics for CsA-CE droplets. Infectious complications were not observed in any of the cases. In a retrospective study^38^, allergic eye disease was a significant predictor of intolerance to Ikervis^®^, with intolerable side effects occurring in 41.5% of patients, whereas in our group no patient discontinued Ikervis^®^ due to intolerable side effects, as in the Vektis study^29^ where the discontinuation rate was 1.8%.

Many studies have found gender predilection, with girls more often affected in BKC^43^ and boys more often affected in VKC^10^. Our study confirms these data.

VKC is mainly a T cell-mediated process, but the persistence of BKC also depends on the T cell response^4,5,27,40,44^. The conjunctival-associated lymphoid tissue and the microbiome together provide immune homeostasis to keep the cornea avascular and transparent while providing a line of defence against the outside world^27,40,44^. Elevated levels of eosinophil cationic protein (ECP), eosinophil neurotoxin (EDN), myeloperoxidase (EPO) and IL-2 are found in the cornea in the setting of either allergic or lid margin inflammation^25^, and CD4+, CD8 + T cells, Th1, Th17 cells and interferon (IFN)-γ, IL-17 A and IL-18 production are downregulated by cyclosporin-mediated calcineurin inhibition^45–47^, resulting in resolution of corneal involvement and subjective symptoms.

The family, general practitioner, pediatrician, and ophthalmologist all have a role to play in determining the management of chronic ocular surface inflammation. Prior to the introduction of CsA-CE treatment, a large number and variety of ophthalmic products were used. Patients had been treated for more than two years before the introduction of CsA-CE drops (Table 1), and the extent of topical steroid treatment was surprisingly high even in cases of moderate VKC and BKC, as the average number of drop bottles in the 3 months prior to CsA-CE initiation was between 3 and 4. Although newer corticosteroids with a better safety profile are available^22^, corticosteroid-induced ocular hypertension and glaucoma, posterior subcapsular cataracts and epithelial toxicity remain significant risks^7,9,16–18^. Our paediatric study cohort demonstrated that the need and amount of topical steroid rescue was almost minimised after initiation of CsA-CE treatment. The steroid-sparing effect of CsA-CE treatment was achieved within the first 3 months and was maintained throughout the 6-month follow-up period.

A limitation of our study was that it lacked a placebo comparator, partly because simple eyewash has a beneficial effect on ocular surface inflammation and partly because it is not ethically acceptable to expose young patients to deteriorating eye conditions without proper treatment. Additionally, the study was conducted at a single centre, which restricts the generalisability of the findings. The authors aim to expand the investigations presented above and to engage readers in the topic by seeking a cooperating partner.

CsA-CE eye drops may be a safe and effective alternative for the long-term treatment of non-infectious chronic ocular surface inflammation with corneal complications in the young population. The introduction of CsA-CE treatment resulted in improvements in both objective and subjective measures. Our study supports the use of CsA-CE 1 mg/ml drops as an early therapeutic approach in young patients, allowing a rapid reduction in corticosteroid use and preventing structural ocular complications.

Serious ocular disease can be largely prevented by early, targeted, safe and effective treatment, and the care of patients with rosacea, eyelid inflammation or allergy can be improved by a multidisciplinary treatment plan.

## Methods

The aim of this prospective study was to evaluate the efficacy of treatment with 0.1% cyclosporin-A (CsA-CE) (Ikervis^®^, 1 mg/ml, Santen Oy, Finland) eye drops in reducing the need for steroids in the treatment of vernal keratoconjunctivitis and non-infectious keratoconjunctivitis associated with blepharitis. The study was conducted at the Department of Ophthalmology, Semmelweis University, in accordance with the tenets of the Declaration of Helsinki and in compliance with the ethical standards set forth by the National Institute of Pharmacy and Nutrition (ethical approval number: KRID: 648905308, OGYÉI/79707-1/2022). Written informed consent was obtained from the parents or legal guardians of each paediatric patient.

We enrolled consecutive paediatric patients with a minimum of six months’ history of moderate to severe perennial vernal keratoconjunctivitis (VKC) or blepharitis-related keratoconjunctivitis (BKC). The study cohort presented with therapy-refractory ocular surface inflammation with corneal involvement, thus initiating CsA-CE treatment. The CsA-CE therapy was approved by the National Centre for Public Health and Pharmacy (NNGYK) in all cases. Patients with ocular pathology other than VKC or BKC (e.g. eyelid anomaly, active or previous herpes simplex, varicella zoster infection, infection or helminthiasis) and those with systemic allergies or ocular or periocular malignancies were excluded from the study.

Subjective ocular symptoms, including tearing, photosensitivity, itching and discharge, were quantified on a visual analogue scale (VAS) ranging from 0 to 10. The maximum score was 40, with the most severe symptom score being^30^. The degree of ocular surface inflammation at baseline and after CsA-CE treatment was graded according to Bonini’s classification of hyperaemic and swollen papillae on the tarsal surface of the eyelid^30^. and corneal and conjunctival fluorescein staining according to the modified Oxford scale^35^.

At baseline, patients diagnosed with VKC or BKC who met the eligibility criteria were administered 1–4 drops of CsA-CE (1 mg/ml) per day, with the dosage determined by their clinical status. Supportive treatments, such as lid care and preservative-free artificial tears, were permitted as required. In the event of clinical deterioration, additional treatment (rescue therapy) was administered for a brief period. This usually consisted of the administration of fluorometholone, hydrocortisone, or dexamethasone drops three to five times daily for a period of five days. The study period encompassed a six-month evaluation of efficacy and overall experience. During the follow-up period, regular check-ups were conducted, during which ocular and general symptoms and signs (tarsal giant papillae, Horner-Trantas dots, keratopathy, neovascularisation, shield ulcer) were recorded.

Patients’ subjective assessments of CsA-CE therapy were rated on a 4-point scale: 0) No effect, (1) Partial effect, (2) Satisfactory and good effect, (3) Rapid and good effect.

The total number of steroid eye drops used was defined as the sum of the number of bottles dispensed in the three months prior to the commencement of CsA-CE treatment. Similarly, the necessity for and quantity of steroid eye drops used by patients were evaluated at three and six months following the initiation of CsA-CE treatment.

The statistical analyses were conducted using the Statistica 14.0.1.25 software (TIBCO Software Inc., Santa Clara, CA, USA). Continuous variables were described in terms of the mean and standard deviation, while discrete variables were described in terms of the number and percentage. A repeated-measures ANOVA was employed to compare the outcome data before and after the introduction of CsA-CE treatment. The data are presented as mean ± standard deviation or percentage, as indicated. A factor was considered to have a significant effect if the p-value was less than 0.05.

## Data Availability

Data availability statementThe datasets generated and/or analysed during the current study may be issued upon request in justified cases. Contact corresponding author.
